# Rational Design of Albumin Theranostic Conjugates for Gold Nanoparticles Anticancer Drugs: Where the Seed Meets the Soil?

**DOI:** 10.3390/biomedicines9010074

**Published:** 2021-01-13

**Authors:** Tatyana V. Popova, Inna A. Pyshnaya, Olga D. Zakharova, Andrey E. Akulov, Oleg B. Shevelev, Julia Poletaeva, Evgenii L. Zavjalov, Vladimir N. Silnikov, Elena I. Ryabchikova, Tatyana S. Godovikova

**Affiliations:** 1 Institute of Chemical Biology and Fundamental Medicine, The Siberian Branch of the Russian Academy of Sciences, Lavrentiev ave. 8, 630090 Novosibirsk, Russia; io197724@gmail.com (T.V.P.); pyshnaya@niboch.nsc.ru (I.A.P.); garonna3@mail.ru (O.D.Z.); fabaceae@yandex.ru (J.P.); silnik@niboch.nsc.ru (V.N.S.); lenryab@niboch.nsc.ru (E.I.R.); 2 Institute of Cytology and Genetics, SB RAS, Lavrentiev ave. 10, 630090 Novosibirsk, Russia; akulov@bionet.nsc.ru (A.E.A.); shevelev@bionet.nsc.ru (O.B.S.); zavjalov@bionet.nsc.ru (E.L.Z.)

**Keywords:** gold nanoparticles, albumin theranostic conjugates, boronated albumin conjugate, trifluorothymidine-albumin conjugate, fluorescence-based molecular imaging

## Abstract

Multifunctional gold nanoparticles (AuNPs) may serve as a scaffold to integrate diagnostic and therapeutic functions into one theranostic system, thereby simultaneously facilitating diagnosis and therapy and monitoring therapeutic responses. Herein, albumin-AuNP theranostic agents have been obtained by conjugation of an anticancer nucleotide trifluorothymidine (TFT) or a boron-neutron capture therapy drug undecahydro-*closo*-dodecaborate (B_12_H_12_) to bimodal human serum albumin (HSA) followed by reacting of the albumin conjugates with AuNPs. In vitro studies have revealed a stronger cytotoxicity by the AuNPs decorated with the TFT-tagged bimodal HSA than by the boronated albumin conjugates. Despite long circulation time, lack of the significant accumulation in the tumor was observed for the AuNP theranostic conjugates. Our unique labelling strategy allows for monitoring of spatial distribution of the AuNPs theranostic in vivo in real time with high sensitivity, thus reducing the number of animals required for testing and optimizing new nanosystems as chemotherapeutic agents and boron-neutron capture therapy drug candidates.

## 1. Introduction

Cancer has become a leading cause of death in industrialized countries [1]. In light of the shortcomings of current treatment modalities for cancer, a critical thrust towards improving cancer therapy is to specifically target therapeutic agents to tumor cells while sparing healthy tissues from harm. This is one of the emerging interests in nanotechnology research. Nanoparticles (NPs) that are most used for cancer nanotechnology include polymers, dendrimers, liposomes, quantum dots, iron oxides, and gold nanoparticles [2]. Among inorganic materials, gold NPs (AuNPs) exhibit unique optical properties, nontoxic nature, and straightforward surface functionalization, providing an attractive platform for cancer theranostics (therapy + diagnostic) development [3,4,5,6,7,8,9]. 

At present, cancer therapy includes surgery, chemotherapy, radiotherapy, targeted therapy, and immunotherapy. AuNPs can destroy cancer cells by photothermal ablation, as exemplified by AuroShell [10], through mechanical damage, or as drug delivery systems for anticancer agents, such as tumor necrosis factor [11], doxorubicin [12], or oxaliplatin [13]. The boron-containing AuNPs represent a new class of therapeutics that can be used for boron neutron capture therapy (BNCT). BNCT is an experimental form of binary radiotherapy that selectively targets and damages tumor cells, even in the very challenging scenario, in which the malignancy is infiltrating into the surrounding normal tissue or is spreading in the whole organ [14,15]. Clinical interest in BNCT has focused primarily on high-grade gliomas [16,17,18,19]. However, boron-containing AuNPs may have limited clinical potential for brain tumors because they are unable to penetrate the blood–brain barrier (BBB) adequately, and they may be trapped in filtrating organs [20]. Therefore, alternative or improved delivery agents are required to implement full clinical potential of BNCT. The delivery of pharmaceuticals through the brain capillary endothelial wall may be facilitated thru conjugation of therapeutics to brain drug delivery vectors. Since human serum albumin (HSA) is shown to undergo absorptive-mediated transcytosis through the BBB in vivo, HSA is a potential brain drug delivery vector in humans [21].

Human serum albumin (HSA) is one of the most popular materials for designing nanoparticles [22,23,24,25,26,27,28]. Since FDA approved Abraxane^®^ (paclitaxel-encapsulated albumin formulation), HSA has attracted increasing attention as a drug cargo. All the factors such as known structure of HSA obtained by X-ray analysis; the ability of the protein to bind and transport various drugs to certain organs of the body; the possibility to obtain recombinant HSA; its great stability at wide range of pH and temperature, as well as in different solvents; the possibility to store the protein in solution for many years make albumin an attractive tool for designing nanocomposite materials to diagnose and treat cancer diseases [22,23,24,25,26,27]. The synergy between HSA and AuNPs results in interesting properties, such as increased nanoparticle stability, their reduced interaction with other plasma proteins, passive and active targeting of the conjugates to malignant cells, and hence increased selectivity [26]. The transcytosis of albumin across the endothelium of blood vessels is achieved through the well-known gp60 pathway which promotes the albumin-bound drugs into tumor cells [29]. Furthermore, active targeting by HSA-conjugated nanosystems involves interaction with several specific receptors, including Secreted Protein Acidic Rich in Cysteine (SPARC). SPARC, which is known as an extracellular matrix glycoprotein acid, is often seen in some neoplasms [30,31]. It has been reported that SPARC is overexpressed in brain tumors, serving as a promoter to glioma progression and invasion, suggesting its potential therapeutic value [32,33].

Nanoparticles with a suitable size to allow for accumulation through passive targeting, if combined with serum albumin, may demonstrate a dual ability to target the malignant cells through passive and active targeting, and therefore represent useful nanoplatforms for drug delivery with significant physiological stability. Authors’ study [28] clearly suggests that stable conjugation of AuNP (core diameter 15 nm) with albumin prior to intravenous administration increases specificity and efficiency of AuNP in diseased target organs thus suggesting a potential role in nanomedicine and nanopharmacology. Therefore, the capping of AuNPs with boronated albumin can improve their biological properties, which may encourage the scientific community to investigate these nanosystems as BNCT drugs, as well as for photothermal therapy.

An important problem in anticancer therapy is to determine biodistribution of the drug carrier after administration. This problem is even more evident when nanosystems are used as boron carriers, as the optimal time window after the drug administration needs to be established for neutron irradiation. For this purpose, equipping the drug carrier with a tag for tracking its location can provide a solution. For example, for nanoparticle-based therapeutic strategies, incorporation of a positron or gamma emitter into the nanoparticle allows for tracking the drug location in a time-resolved fashion using positron emission tomography (PET) or single photon emission computerized tomography (SPECT) imaging [34,35]. However, these methods have their limitations. While PET is useful for clinical applications due to its ability to provide information about spatial distribution of drugs in vivo, its spatial resolution of a millimeter scale makes it difficult to reveal precise drug location. Autoradiography enables subcellular spatial resolution for distribution of boron drugs, while it requires a neutron beam source for triggering nuclear reaction, which is not always available.

Among many detection modalities, ^19^F MRI is advantageous for deep-tissue and noninvasive imaging in vivo [36,37,38]. In vivo experiments using the C6 rat glioma model demonstrated that ^19^F MRI in combination with ^1^H MRI can selectively map the biodistribution of a ^19^F-labelled borophenylalanine-fructose complex [39]. The NMR sensitivity of ^19^F is 0.83 relative to ^1^H, has a 100% natural isotopic abundance ratio, and has a large chemical shift range (300 ppm). In addition, the human body itself provides a negligible endogenous ^19^F MRI signal [36]. 

Previously [38], we have developed a novel anticancer albumin-trifluorothymidine theranostic conjugate PFT-Hcy-HSA-Cy7-pTFT. The in vivo antitumor effect of this albumin theranostic agent was evaluated on a U87 glioma-bearing mouse model [40]. The results clearly demonstrated that the albumin theranostic can significantly suppress the tumor. Our initial results demonstrate the potential of PFT-Hcy-HSA-Cy7-pTFT conjugate to serve as an optical and ^19^F MRI magnetic resonance imaging agent [38]. Based on their detection sensitivity, the combination of ^19^F MRI and fluorescence imaging is probably the most valuable one in multimodal molecular imaging.

Here, we report on the preparation and characterization of AuNPs stabilized with dual-labeled albumin, containing fluorophore and ^19^F tags (bimodal serum albumin), which is functionalized with the chemotherapeutic agent (trifluorothymidine) or boron-rich anion (undecahydro-*closo*-dodecaborate). The albumin theranostic conjugates allows for direct optical and ^19^F magnetic resonance imaging [38]. Fluorescence-based molecular imaging becomes increasingly important, mainly due to the development of highly sensitive cameras and very high spatial resolution. We used this modality in combination with computed tomography to generate anatomical details of an animal subject studied after albumin-AuNP conjugate to intravenous administration.

## 2. Experimental Section

### 2.1. Materials

Reagents and materials were purchased from Sigma-Aldrich (UK), unless otherwise indicated. Milli-Q water with conductivity greater than 18 MΩ/cm was used in all experiments. Gold (III) chloride trihydrate (HAuCl_4_ 3H_2_O) and sodium citrate solution (Sigma–Aldrich Chem. Co., St. Louis, MO, USA) were used as received. HSA (A3782) was also obtained from Sigma–Aldrich Chem. Co. (St. Louis, MO, USA). 

Dual-labeled albumin was synthesized according to the published procedure [37] and kindly provided by Dr. Alexey S. Chubarov (Institute of Chemical Biology and Fundamental Medicine, SB RAS, Lavrentiev ave. 8, Novosibirsk, 630090, Russia).

Maleimide-functionalized *closo*-dodecaborate (MID-B_12_H_12_) was synthesized according to the published procedure [41] and kindly provided by Dr. Ludmila S. Koroleva (Institute of Chemical Biology and Fundamental Medicine, SB RAS, Lavrentiev ave. 8, Novosibirsk, 630090, Russia).

Maleimide functionalized of 5-trifluoromethyl-2’-deoxyuridine 5’-monophosphate was synthesized according to the published procedure [38] and kindly provided by Dr. Vladimir A. Lisitskiy (Institute of Chemical Biology and Fundamental Medicine, SB RAS, Lavrentiev ave. 8, Novosibirsk, 630090, Russia).

### 2.2. Instrumentation

The absorption spectra of colloidal solutions of AuNPs in quartz cells (1 cm path length) were registered on a Shimadzu UV-2100 spectrophotometer (Shimadzu, Japan). The concentrations of albumin solutions were determined by absorption at 292 nm, pH 13, using the molar extinction coefficient *ε* = 4.44 × 10^4^ M^-1^cm^−1^ [42] with a UV-1800 spectrometer (Shimadzu, Japan). 

To determine hydrodynamic diameter and zeta-potential of all NPs used in the study, we applied a Zetasizer Nano ZS Plus instrument (Malvern Instruments; Malvern, Worcestershire, UK). All measurements were made in triplicate and according to manufacturer instruction.

For electron microscopic characterization, suspensions of all studied AuNPs were adsorbed for 30 s on copper grids coated with formvar film, which was stabilized by carbon using a JEE-420 Vacuum Evaporator (Jeol, Tokyo, Japan). Then, excess liquid was removed and samples were negatively stained with phosphotungstic acid (pH 3.0) for 10 s. The grids were examined in a JEM 1400 (JEOL, Tokyo, Japan) electron microscope at an accelerating voltage of 80 kV, images were collected using a Veleta digital camera (EM SIS, Munster, Germany). The sizes of AuNPs were measured using the iTEM program, version 5.2 (EM SIS, Munster, Germany), on the camera screen. 

^1^H and ^19^F NMR spectra were recorded on AV-300 NMR spectrometer (Bruker, Germany) at 300.13 and 282.7 MHz, (^1^H and ^19^F NMR spectra, respectively). The spectra were detected in 5 mm NMR sample tubes at 25 °C. The chemical shifts were expressed in parts per million (ppm, *δ*). Residual ^1^H NMR signal of the deuterated NMR solvent (D_2_O, *δ* 4.80 ppm) was used as a reference for all ^1^H chemical shifts. Perfluorobenzene (*δ* 0.00 ppm) was used as an external reference for chemical shifts in ^19^F NMR spectra. ^31^P NMR spectra were recorded on AV-400 and AV-300 NMR spectrometers (162 and 121 MHz, respectively). The ^31^P chemical shifts were calculated using an external standard of 85% H_3_PO_4_ (*δ* 0.00 ppm). The spin–spin coupling constants (*J*) are reported in hertz (Hz), and spin multiples are given as s (singlet), d (doublet), t (triplet), and m (multiplet). Broad peaks are indicated by the addition of br.

Low-molecular-weight materials (MW < 3 kDa) were removed from solutions of polymer conjugates by centrifugal filtration using Centricon concentrators with a MWCO of 3-kDa (Amicon Centriprep YM30, Millipore, Bedford, MA, USA).

### 2.3. Synthesis and Characterization of Theranostic Conjugate PFT-Hcy-HSA-Cy7-pTFT

The PFT-Hcy-HSA-Cy7-pTFT conjugate is a known HSA conjugate. The synthetic procedure of theranostic conjugates PFT-Hcy-HSA-Cy7-pTFT was performed out in the dark according to the method described elsewhere [38].

The yield of PFT-Hcy-HSA-Cy7-pTFT was 50%. ^19^F NMR (PBS buffer, pH 7.4, *δ*, ppm): 108.0 (br m, CF_3_, perfluorotoluene residues), several overlapping signals at 100.0 ppm (m, CF_3_, 5-C). ^31^P NMR (D_2_O, *δ*, ppm): 8.89. UV–VIS (PBS buffer, pH 7.4): *λ*_max_ nm (*ε*, M^-1^cm^-1^): 267 ((1.4 ± 0.1) × 10^5^), 292 ((1.35 ± 0.1) × 10^5^), and 762 ((1.8 ± 0.1) × 10^5^). Fluorescence: the excitation and emission maximum wavelengths are 762 nm and 780 nm, respectively.

### 2.4. Synthesis and Characterization of Theranostic Conjugate HSA-Cy5-Hcy-TFAc-B_12_H_12_

The synthesis of HSA-Cy5-HcyTFAc conjugate was adapted from Lisitskiy et al. [38]. Briefly, a solution (0.5 mL, 0.7 mM, 0.35 μmol) of dual-labeled albumin (HSA-Cy5-HcyTFAc) in PBS buffer (pH 7.4) was mixed with MID-B_12_H_12_ in DMSO (29.2 μL, 1.58 μmol, 0.166 M). The reaction mixture was incubated under constantly gently stirring at 37 °C in the dark for 17 h. The protein conjugates were purified by SEC utilizing a Millipore ultrafiltration tube and stored at 4 °C.

The yield of HSA-Cy5-HcyTFAc-B_12_H_12_ conjugate was ~65%. UV–VIS (PBS buffer, pH 7.4): *λ*_max_ 278 nm (*ε* = (3.88 ± 0.1) × 10^4^) and *λ*_max_ 646 nm (*ε* = (2.7 ± 0.1) × 10^5^). Inductively coupled plasma atomic emission spectroscopy: 2.6 B_12_H_12_ residues per albumin. 

### 2.5. Synthesis of Gold Nanoparticles

Gold nanoparticles (AuNPs) were prepared using citrate method [43]. In brief, 5 mL (38.8 mM) of sodium citrate solution was added to 45 mL (1.1 mM) of HAuCl_4_ 3H_2_O boiling water solution under intense stirring. The mixture was stirred for 20 min and then kept for 24 h at room temperature, after that, the suspension was passed through a 0.45 µm filter (Millipore, USA) and stored at 4 °C. According to TEM data, the obtained AuNPs had a spherical shape and a diameter of 12 ± 0.1 nm. Hydrodynamic diameter of AuNPs was 17.40 ± 0.40 nm, zeta potential was −33.60 ± 2.04 mV, and the maximum absorption spectrum was 520 nm.

### 2.6. Synthesis and Characterization of Multifunctional Human Serum Albumin-Therapeutic Nucleotide Conjugates alb-TFT-AuNP and alb-boron-AuNP 

To obtain albumin-modified AuNPs, citrate-AuNPs (0.9 mL of 1.16 nM) were incubated with conjugates PFT-Hcy-HSA-Cy7-pTFT and HSA-Cy5-Hcy-TFAc-B_12_H_12_ (0.1 mL of 0.1 mM) in PBS for 24 h at room temperature. The reaction mixtures were centrifuged for 15 min (13,000 rpm) to remove unbound albumin conjugates. The pellets were resuspended in PBS and kept at 4 °C. The alb-TFT-AuNP had a zeta potential of −32.05 ± 4.70 mV and a hydrodynamic diameter of 25.93 ± 0.60 nm; the diameter measured by TEM was 17.55 ± 1.14 nm. The alb-boron-AuNP had a zeta potential of −22.43 ± 1.12 mV and a hydrodynamic diameter of 28.93 ± 0.08 nm; the diameter measured by TEM was 17.25 ± 1.58 nm.

The nanoparticle suspensions in PBS were kept at 4 °C and application was done in the in vitro and in vivo studies within 3–10 days. Prior to tail vein injection, zeta-potential and hydrodynamic diameter of albumin-AuNP conjugates were determined by dynamic light scattering measurements. Noteworthy, the physicochemical parameters of the used AuNPs conjugates remained unaltered during storage.

### 2.7. Cell Viability Assay (MTT Test)

Human mammary adenocarcinoma cell line MCF-7 was cultured in IMDM medium (Invitrogen) supplemented with 10% fetal bovine serum (FBS) (Invitrogen), penicillin (100 units/mL), and streptomycin (100 μg/mL) at 37 °C and 5% CO_2_ in humid atmosphere.

The inhibition of cell growth was assessed by MTT test, based on the reduction in MTT (3-(4,5-dimethylthiazol-2-yl)-2,5-diphenyltetrazolium bromide into formazan by mitochondrial NAD(P)H-dependent oxidoreductase enzymes [44]. Cells in exponential growth phase were seeded in 96-well plates (2000 cells per well). The cells were allowed to attach for 24 h and were treated with HSA, alb-boron-AuNP, alb-TFT-AuNP, HSA-Cy5-Hcy-TFAc-B_12_H_12_, PFT-Hcy-HSA-Cy7-TFT, or pTFT. The solutions of albumin conjugates were applied in medium with HSA-equivalent concentrations ranging from 0.06 to 60 µM for 72 h at 37 °C. MTT solution was added to each well at final concentration of 0.5 mg/mL, and the plates were incubated at 37 °C for 2 h. The medium was removed and the dark blue crystals of formazan were dissolved in 100 μL of isopropanol. The absorbance at 570 nm (peak) and 620 nm (baseline) was determined using a microplate reader Multiscan FC (Thermo Fisher Scientific Corporation). All concentrations were performed in triplicate. The values are given as mean ± standard deviation (S.D.) values, and all measurements were repeated three times.

### 2.8. Animal Care, Maintenance of Tumors, and Experimental Procedures

The trials with the mice of the SCID line were performed at the Center for Genetic Resources of Laboratory Animals at the Institute of Cytology and Genetics, Siberian Branch, Russian Academy of Sciences (RFMEFI62119X0023). All animal experiments described in this paper were carried out in compliance with the principles of humanity according to directive 86/609/EEC of the European Community. All efforts were made to minimize the number of animals used and their suffering. 

The study was conducted on 8 male mice of the SCID (SHO-PrkdcscidHrhr) line of the SPF status at the age 6–7 weeks. The animals were kept in sn SPF-vivarium of the Institute of Cytology and Genetics, Siberian Branch, Russian Academy of Sciences (SB RAS) in unisexual family groups of 4 individuals in individually ventilated cages (IVC) of the OptiMice system (Animal Care Systems) under the controlled conditions (at a temperature of 22–26 °C, relative humidity of 30–60%, and light mode light/dark 14/10 h with dawn at 1:00 a.m.). The Sniff feed (Germany) and deionized water enriched by Severyanka mineral mixture (St. Petersburg) were given to the animals ad libitum.

U87 line of glioma cells were used in the experiments [45]. About 2–3 weeks before the beginning of the experiment, the U87 glioma cell culture, which is stored in the cryobank of the Center of Collective Use SPF-vivarium of the Institute of Cytology and Genetics SB RAS in liquid nitrogen, is defrosted and cultivated for 5–7 passages on a DMEM/F12 medium (1:1) with a 10% fetal serum (FBS) produced by Invitrogen. Before the injection, the tumor cells were removed from the substrate by a tripsin/versene solution, and the precipitate was thoroughly resuspended in the medium without a serum after centrifuging for 5 min at 1000 revolutions per min (bringing to the concentration 100,000 cells in 1 μL). 

Orthotopic xenotransplantation of U87 cells is done into mice of the SCID line. Before the operation, the animal was placed in the chamber with the air inflow of 450–500 mL/min and isoflurane concentration in the air mixture of 1.5%. After 3 min, the animal was transferred onto a heated operating table with a surface temperature of 37 °C and placed under an anesthetic mask with an isoflurane in oxygen mix (1.5%, flow rate 200 mL/min) for anesthesia. The mice (n = 8) received incranial injection of inocula of 300 × 10^3^ tumor cells at left hemisphere near the hippocampus (-4 mm Bregma). The cell suspension was introduced in the subcortical brain structures through a hole in the animal’s cranium. For this, a skin incision 3–4 mm in length was made on the head in the caudal–cranial direction near the bregma, and 5 μL of the cell suspension was introduced through the hole in the cranium.

Before FLECT/CT scanning, animals were immobilized using 75 µL/10 g body weight of Dorbene (Laboratories SYVA S.A., Leon, Spain) and 5 min later, 80 µL/10 g body weight of Zoletil (Virbac Sante Animale, Carros, France).

### 2.9. FLECT/CT

Mice were imaged in InSyTe FLECT/CTTM system (TriFoil Imaging, Chatsworth, CA, USA). CT was performed using next parameters: tube voltage 35 kV, tube current 950 µA, and exposure time 180 ms. The entire object was scanning, according to the principle of continuous helical radiation of 720 projections with 360-degree coverage. Image resolution reaches 25 × 25 × 25 microns. Total time of study was 15 min. FLECT was performed using the 730 nm laser, and the fluorescence signal was filtered with 813 nm filter emission. Exposure time was 16 msec. The entire object was scanning, according to the principle of continuous helical radiation of 116 projections with 360-degree coverage. Image resolution reaches 1 mm × 1 mm × 1 mm. Total time of study was 40 min. The images were reconstructed using the TriFoil Imaging software (TriFoil Imaging, Chatsworth, CA, USA). CT and FLECT reconstructions were combined using VivoQuant v2.5 (Invicro, Boston, MA, USA).

The investigation of PFT-Hcy-HSA-Cy7-pTFT and alb-TFT-AuNP distributions in SCID mice with the brain tumor caused by the intracranial injection of a human glioblastoma cell line U87 were held 4 weeks after the injection of tumor cells. Before FLECT/CT scanning, 0.1 mL of 0.1 mM solution of albumin conjugates was intravenously injected in the mouse. The mice were imaged by InSyTe FLECT/CTTM system in vivo over time after injection of albumin conjugates (at 1 and 72 h after intravenous injection).

## 3. Results and Discussion

### 3.1. Synthesis of Functionalized AuNPs 

Here, based on the previous works [46,47,48,49], we envisaged the possibility of using gold nanoparticles (AuNPs) as drugs carrier. Besides easy functionalization chemistry, gold nanomaterials are chemically inert and nontoxic, and both their shape and size can be easily tuned [47]. Among all the possibilities, we decided to start with spherical particles with a size within the optimal range (sizes larger than 10 nm) to achieve tumor accumulation [48]. Here, colloidal AuNPs were prepared by the Turkevich-Frens method using trisodium citrate as a reducing agent in water [43]. AuNPs showed their characteristic SPR (surface plasmon resonance) peak at 521 nm. Transmission electron microscopy (TEM) images revealed spherical and monodisperse particles in the size range of 11–13 nm. DLS (Dynamic Light Scattering) particle size distribution indicated an average particle size of 17.40 ± 0.40 nm.

**Figure 1:**
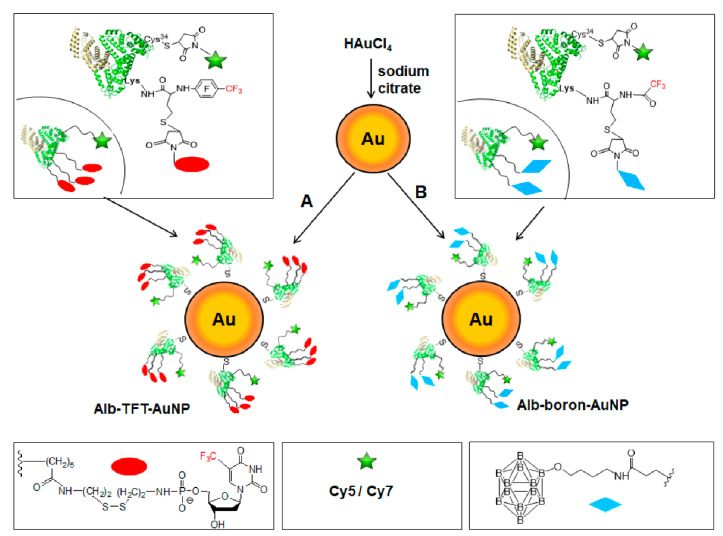
Design of multimodal albumin-gold nanoparticles (AuNPs) theranostic constructs—alb-TFT-AuNP (**A**) and boronated conjugate alb-boron-AuNP (**B**). A protein carrier human serum albumin (HSA) is shown with its crystal structure in ribbon representation. An effector—a chemotherapeutic agent pTFT—is depicted as a red ellipse. Note that pTFT is conjugated with HSA via a linker connecting the 5’-phosphate group of pTFT with ε-amino groups of protein’s residues Lys-525, Lys-205, and Lys-137, which were identified as primary conjugation sites [40]. boron neutron capture therapy (BNCT) agent—undecahydro-close-dodecaborate—is shown as a blue rhombus. The fluorine-labeled homocysteine residues (PFT-Hcy and TFA-Hcy) are used as functional handles (linkers). CF_3_-based ^19^F-MR imaging is enabled by the presence of fluorinated compounds (trifluorothymidine (TFT) and trifluoroacetate and perfluorotoluene residues). Optical imaging is achieved due to conjugation of fluorescent dyes Cy7/Cy5 (green star) to Cys-34 residue of HSA.

Functionalized AuNPs (alb-TFT-AuNP and alb-boron-AuNP) were prepared by mixing spherical citrate-stabilized AuNPs with theranostic conjugates PFT-Hcy-HSA-Cy7 pTFT (Figure 1A) and HSA-Cy5-Hcy-TFAc-B_12_H_12_ (Figure 1B).

**Scheme 1:**
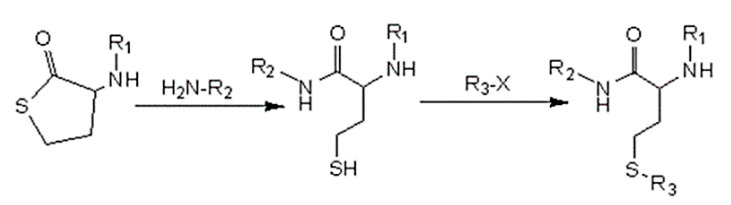
A thiolactone entity as a latent thiol functionality: the thiol is released by nucleophilic ring-opening of the cyclic thioester for a subsequent thiol click reaction to incorporate the R_3_ residue. ^19^F-MR imaging (R_1_)—fluorinated compounds (trifluoroacetate/perfluorotoluene residues). Drug carrier (R_2_)—human serum albumin (HSA)-Cy5/Cy7. Therapeutic agent (R_3_)—maleimide-functionalized undecahydro-close-dodecaborate or a chemotherapeutic agent TFT.

For the synthesis of an albumin-based theranostic agents PFT-Hcy-HSA-Cy7-pTFT and HSA-Cy5-Hcy-TFAc-B_12_H_12_, we used reactivity of a thiolactone (a cyclic thioester) as a latent thiol functionality in thiol-“click” chemistry. The thiol was released by nucleophilic ring-opening (aminolysis) by amino groups on the HSA and subsequently reacted with a thiol “scavenger” (a maleimide derivative of the drug) according to the Scheme 1. Detailed description of the synthetic procedure of albumin theranostic agents can be found in [38]. The chemical structure of the obtained conjugates was confirmed by ^19^F NMR, ^31^P NMR, and UV–VIS spectroscopy (Figures S1 and S2, ESI†).

**Figure 2:**
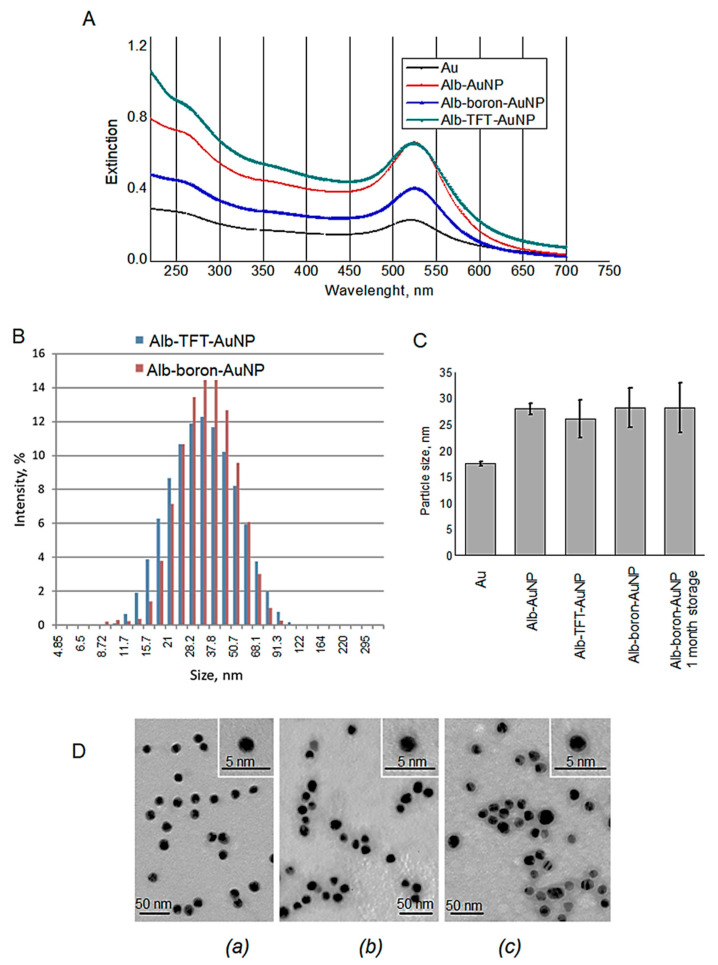
(**A**) UV–VIS spectra corresponding to citrate-stabilized AuNPs: AuNPs-citrate, black, *λ*_max_ = 521 nm; human serum albumin (HSA)-Au, red, *λ*_max_ = 525 nm; alb-TFT-AuNP, green, *λ*_max_ = 524 nm and alb-boron-AuNP, blue *λ*_max_ = 525 nm for the gold plasmon; *λ*_max_ = 260 nm corresponds to compound TFT; (**B**) size distribution ranges as determined by dynamic light scattering (DLS) (volume distribution) for alb-TFT-AuNP, and alb-boron-AuNP; (**C**) particle size of AuNPs; (**D**) representative TEM images of AuNPs-citrate (*a*), alb-TFT-AuNP (*b*) and alb-boron-AuNP (*c*). Negative staining with phosphotungstic acid (pH 3.0). Bars correspond to 5 nm (inset) and 50 nm (main image).

Simultaneous modification of AuNPs with trifluorothymidine and HSA resulted in a bathochromic shift of the longitudinal SPR band from 521 to 524 nm (Figure 2A). Additionally, another absorption band appeared at about 260 nm, close to the maximum absorption of TFT (267 nm), confirming successful incorporation of the TFT on the surface of the AuNPs. The amount of undecahydro-*closo*-dodecaborate in the final alb-boron-AuNP conjugate determined by inductively coupled plasma atomic emission spectroscopy (ICPE-9820, Shimadzu, Japan) was estimated to be about 0.307 µg of boron/mg of gold.

Zeta potential of a nanoparticle characterizes its surface charge, and magnitude of the potential depends on a composition of both nanoparticles and solvent in which they are suspended. alb-TFT-AuNP and alb-boron-AuNP have a negative zeta potential of −32.05 ± 4.7 and −22.43 ± 1.12 mV at neutral pH, respectively. The zeta potential could serve as a parameter for predicting the stability of the NP suspensions, i.e., its value above 30 mV (both positive and negative) is typical for stable suspensions. It is believed that the repulsive forces arising from surface charges can prevent the aggregation of particles. The stability test was undertaken using the sample alb-boron-AuNP stored at 4 °C for 30 days. No changes in the morphology of nanoparticle-based conjugates before and after storage was observed by TEM, suggesting good stability. 

An important issue in the design of nanoparticles is their pharmacokinetic properties [44]. In this respect, size and charge are key determinant factors. Large particles are more prone to be cleared by the reticuloendothelial system. Therefore, particle sizes below 100 nm are desirable. Dynamic light scattering (DLS) analysis performed on alb-TFT-AuNP and alb-boron-AuNP showed a mono-disperse distribution with average hydrodynamic diameter of 25.93 ± 0.60 and 28.08 ± 1.79 nm, respectively (Figure 2B,C). TEM analysis revealed rare loose aggregations and single particles (Figure 2D). Gold core of the NPs had diameter of 12.24 ± 0.97 nm and was covered with a layer of middle electron density (Figure 2D), having the thickness of 2.26 ± 0.27 and 2.82±0.38 nm, correspondingly. The size distribution in both groups is in accordance with the DLS results (Figure 2C), though the diameters measured by DLS are slightly higher than the ones by TEM. This discrepancy is expected, since TEM measures the accurate solid spheres, whereas DLS approach gives the hydrodynamic diameters.

### 3.2. In Vitro Study

**Figure 3:**
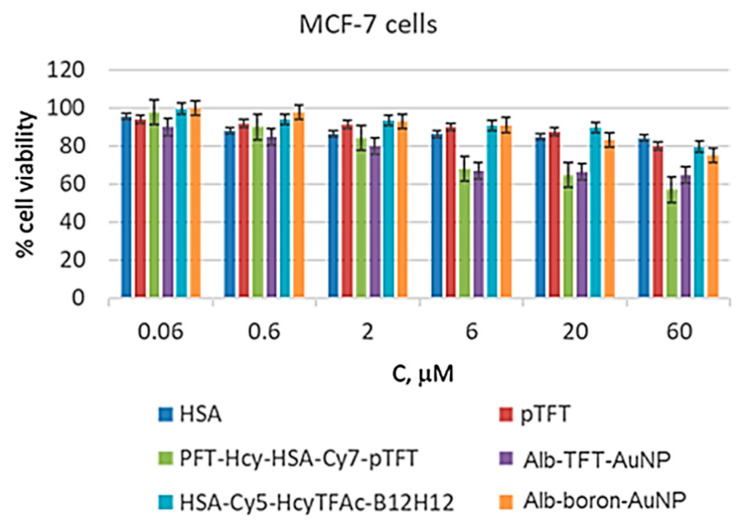
In vitro cytotoxicity of all formulations evaluated using MCF-7 cells by MTT assay [44] (n = 3). The MCF-7 cell line was incubated for 72 h with HSA (dark blue), pTFT (red), PFT-Hcy-HSA-Cy7-pTFT (green), alb-TFT-AuNP (violet), HSA-Cy5-Hcy-TFAc-B_12_H_12_ (cyan), and alb-boron-AuNP (orange) loaded at various concentrations. Cell viability was normalized using the cells treated with a PBS buffer as a 100% viability control.

PFT-Hcy-HSA-Cy7-pTFT conjugate have been shown to be active against several cell lines [38]. Here, treatment of the MCF-7 cells with free pTFT resulted in high cell viability (around 80%) at the maximum concentration of free pTFT (Figure 3). 

However, when the HSA conjugates containing pTFT were employed, viability of the MCF-7 cells decreased in a dose-dependent manner, reaching 65% and 57% at 60 μM of the conjugates alb-TFT-AuNP and PFT-Hcy-HSA-Cy7-pTFT, respectively. The drug-free carrier HSA, at the same time, exhibited a very low cytotoxic activity (e.g., 80% cell viability at 60 μM after 72 h). Alb-TFT-AuNP showed the superior pharmaceutical activity relative to the drug-free carrier. It can be attributed to the release of the active drug pTFT from the conjugate in the tumor cells. These results confirm the suggestion that albumin is an attractive candidate for incorporation into drug delivery systems. 

The cytotoxicity analysis revealed the nontoxic nature of the boronated albumin theranostics, as both HSA-Cy5-Hcy-TFAc-B_12_H_12_ and alb-boron-AuNP did not trigger substantial loss of MCF-7 cell viability even at high conjugate concentrations (Figure 3). Indeed, the lowest toxicity observed for both boronated conjugates among other conjugates studied, and a proliferation rate of over 85% was observed within the conjugate concentration range of 0.06–20 μM. Thus, for neutron source efficacy evaluation, 20 μM boronated albumin theranostics can be used to minimize the effect of the drug on glioma cell colony formation. Conjugation of boron compounds and HSA—a carrier protein with a long plasma half-life—is expected to extend systemic circulation of boron compounds and preserve its activity. Boron–albumin conjugates are being developed for the treatment of cancer patients to provide a sustained exposure that can reduce frequency of injections and consequently improve compliance and quality of life for patients.

### 3.3. In Vivo Imaging Studies 

**Figure 4:**
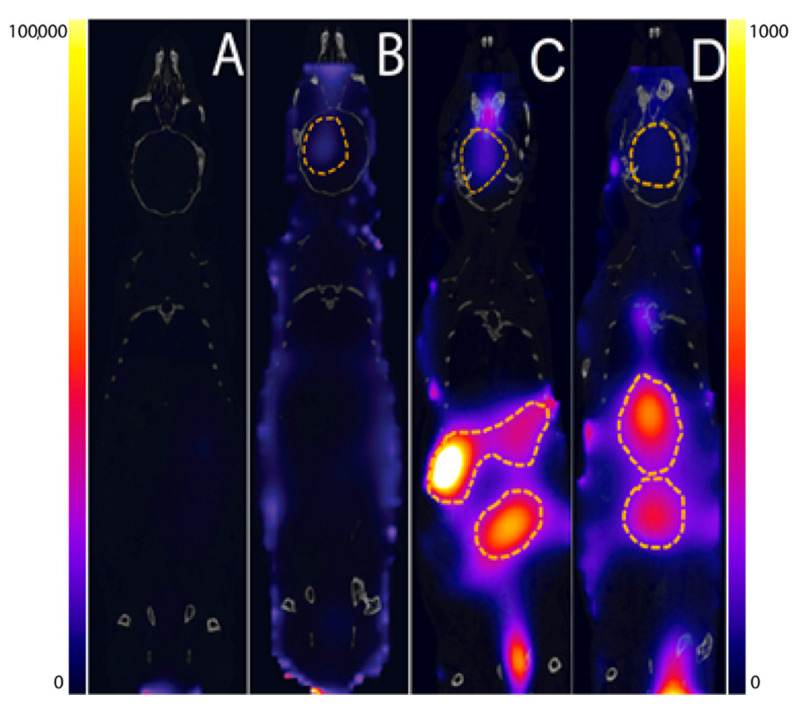
The whole body FLECT (Fluorescence Emission Computed Tomography)/CT (Computed Tomography) images of PFT-Hcy-HSA-Cy7-pTFT (**A**,**B**) and alb-TFT-AuNP (**C**,**D**) distribution at different time points in mice with severe combined immunodeficiency (SCID) with the brain tumor caused by the intracranial injection of a human glioblastoma cell line U87. Images were taken under the same conditions at 1 h (**A**,**C**) and 72 h (**B**,**D**) postinjection. Albumin conjugate PFT-Hcy-HSA-Cy7-pTFT accumulates in the malignant tissue (dotted line) of mouse brain 72 h after their intravenous injection (**B**). alb-TFT-AuNP conjugate predominantly localizes in liver (**C**,**D**). For a better visual display of the fluorescent label distribution in animals (**A**,**B**) and (**C**,**D**), different boundary conditions of the signal intensity scale were chosen.

Our labelling strategies enable determination of the nanoconjugate location in vivo, in real time and with high sensitivity, thus reducing the number of animals required for fast investigation of new nanosystems as chemotherapeutic agent and BNCT drug candidates. Here, imaging studies performed in mice enabled determination of the biodistribution pattern of the labelled HSA conjugates and functionalized AuNPs (Figure 4). 

To do this, we used fluorescence-based molecular imaging in combination with computed tomography (CT) to obtain anatomical details of the studied animal. The tests were carried out on SCID SPF mice with a brain tumor caused by intracranial injection of the U87 human glioblastoma cell line [45]. The xenograft-carrying SCID mice were injected intravenously with albumin conjugate at a pTFT concentration of 10^-4^ M. Whole body fluorescence images were recorded 1 and 72 h after injection using the InSyTe FLECT/CT system (TriFoil Imaging, Chatsworth, CA, USA) (Figure 4). FLECT/CT system allows capturing true 3D tomographic images by acquiring projection images 360° around the animal subject and sequent accurate image reconstruction. 

These initial animal experiments in a glioma model demonstrated a marked accumulation of the fluorescent signal of the PFT-Hcy-HSA-Cy7-pTFT conjugate in the tumor lesion after 72 h postinjection (Figure 4B). Unfortunately, our alb-TFT-AuNP did not show significant accumulation in the tumor. It has been found that, upon single administration, alb-TFT-AuNP conjugate predominantly localizes in liver, with maximal accumulation in the tumor achieved at 1 h after administration (Figure 4C). Such accumulation progressively decreased with time and is almost undetectable at 72 h (Figure 4D). 

AuNPs biodistribution depends on such factors as size, surface change, and surface functionalization [28,50]. It is known, that 15- and 50-nm AuNPs can pass the blood-brain barrier, as evident from gold concentration in brain [50]. Interestingly, 15-nm AuNPs cause higher accumulation of gold in all the tissues including blood, liver, lung, spleen, kidney, brain, heart, stomach than the bigger particles. While these studies imply that the particle size plays a key role in biodistribution, such factor as surface chemistry, which has significant effect on the NPs interactions with biological systems, was ignored in these studies. Until now, it is still not clear whether the same-sized AuNPs coated with different surface ligands have different biodistribution and accumulation in tissues. Surprisingly, conjugation of AuNPs with human albumin (alb-AuNP) increases blood circulation time and significantly enhances translocation of the particles into brain [28]. Histologically, the 15-nm alb-AuNPs are mainly located in the endothelium of brain, lungs, liver and kidneys after 30 min, while at 19 h, they move deeper into the parenchyma [28]. The concept of attaching ligands to the NPs so as to allow them to hone to the tumor appears logical and simple but is, in fact, fraught with difficulties. Our results indicate that the attachment of the modified albumin to AuNP can affect the functioning of AuNP-based drugs and, therefore, should be taken into consideration when designing the conjugates with desirable pharmaceutical properties.

Our group has previously reported CT scans on PFT-Hcy-HSA-Cy7-pTFT [38]. Furthermore, we showed that conjugation of the drug to the protein led to its increased accumulation in the tumor. In the present work, we have demonstrated, for the first time, significant difference in bioaccumulation and retention pattern of the alb-TFT-AuNP conjugate in various organs with much less tumor accumulation, and significant accumulation of the conjugates in the liver when compared to the control PFT-Hcy-HSA-Cy7-pTFT. However, it is not clear whether the uptake of the drug in the liver is due to either recognition of the albumin-AuNP by macrophages and its phagocytosis or receptor-mediated uptake by cells with high expression of receptors to the modified albumin. 

We speculate that higher retention of alb-TFT-AuNP in the liver is due to the protein denaturation within the AuNP conjugate. HSA contains a single cysteine residue—Cys34—and 17 disulfide bridges [42]. The thiol group in albumin can be easily attached to the surface of AuNPs to form a stable S-Au bond [51]. However, the fluorescent tag was introduced into the alb-TFT-AuNP conjugate via a reaction between maleimide Cy5 and the albumin’s single cysteine, Cys34. Therefore, the free sulfhydryl group necessary for the protein’s interaction with AuNP is blocked in alb-TFT-AuNP. The disulfide bonds of HSA are located in different places; some are on the surface and, therefore, can easily contact Au. Meanwhile, the formation of the S-S-Au bond may cause changes in the secondary structure of HSA [51]. Better understanding of modified albumin–receptor interactions would enable a more rational approach to the design of albumin conjugates with a long half-life without compromising its anticancer property.

Although poor tumor accumulation was achieved, appropriate redesign of the albumin-AuNP core in terms of size and shape should lead to improved results. Compared to nanospheres, gold nanorods are known for their lower uptake by the liver, longer circulation time in the blood, and higher accumulation in the tumors [52]. We anticipate that the use of nanorod-shaped particles should lead to lower sequestration by the liver, as previously reported [52]. This would hopefully increase the conjugate’s circulation time and facilitate its accumulation in the tumor. Additionally, gold nanorods exhibit the well-known ability to absorb near infrared (NIR) light, enabling photothermal therapy and thus paving the way towards combined therapies.

## 4. Conclusions

Theranostic AuNPs combine diagnosis with therapy of malignant cells in the same platform for convenient application in vitro and in vivo. In the present work, we described the preparation and characterization of size and shape-tuned of two novel conjugates based on gold nanoparticles (AuNP), fluorine-labeled albumin, fluorescent dye, and with decorated drugs/agents involved in cancer therapy. The first alb-TFT-AuNP conjugate contains trifluorothymidine (TFT) as chemotherapeutic agent, while the second alb-boron-AuNP conjugate include undecahydro-*closo*-dodecaborate. A theranostic agent alb-TFT-AuNP could efficiently kill the tumor cell. At the same time, alb-boron-AuNP did not reveal any significant reduction in cell viability with respect to the parent albumin.

Our unique labelling strategy allows for monitoring of spatial distribution of the AuNPs theranostic in vivo in real time with high sensitivity. This study demonstrates, for the first time, a severely different accumulation and retention pattern in various organs with much less tumor accumulation and significant accumulations in liver the albumin-AuNP-conjugate when compared to the control PFT-Hcy-HSA-Cy7-pTFT. These results provide fundamental insights into the effects of nanoparticles on the adsorbed protein structure and a somewhat cautionary note about selecting nanoparticle-based applications in bioapplications. Thus, improving functional design and exploiting new structure of AuNPs remain essential for cancer theranostics.

## Data Availability

The funders had no role in the design of the study; in the collection, analyses, or interpretation of data; in the writing of the manuscript, or in the decision to publish the results.

## Supplementary Materials

The following are available online at https://www.mdpi.com/2227-9059/9/1/74/s1, Figure S1: Characterization of multifunctional human serum albumin-therapeutic conjugate HSA-Cy5-HcyTFAc-B_12_H_12_ and Figure S2: Characterization of multifunctional human serum albumin-therapeutic conjugate PFT-Hcy-HSA-Cy7-pTFT.
