# Supplementary material for: Rational Design of Albumin Theranostic Conjugates for Gold Nanoparticles Anticancer Drugs: Where the Seed Meets the Soil?
Source: Biomedicines. 2021 Jan 13;9(1):74. doi: 10.3390/biomedicines9010074 (PMC7828547; doi:10.3390/biomedicines9010074)
Supplement: Supplementary file 1 [file biomedicines-09-00074-s001.pdf]

Supporting information

for

Rational design of albumin theranostic conjugates for gold nanoparticles anticancer drugs: where the seed meets the soil?

Tatyana V. Popova[1], Inna A. Pyshnaya[1], Olga D. Zakharova,[1] Andrey E. Akulov[2], Oleg B. Shevelev[2], Julia Poletaeva[1], Evgenii L. Zavjalov[2], Vladimir N. Silnikov[1], Elena I. Ryabchikova,[1] and Tatyana S. Godovikova[1]

[1] Institute of Chemical Biology and Fundamental Medicine, The Siberian Branch of the Russian Academy of Sciences, Lavrentiev ave. 8, Novosibirsk, 630090, Russia; io197724@gmail.com (T.V.P.), pyshnaya@niboch.nsc.ru (I.A.P.), garonna3@mail.ru (O.D.Z.), fabaceae@yandex.ru (J.P.), silnik@niboch.nsc.ru (V.S.), lenryab@niboch.nsc.ru (E.R.)

[2] Institute of Cytology and Genetics, SB RAS, Lavrentiev ave. 10, Novosibirsk, 630090, Russia; akulov@bionet.nsc.ru (A.E.A.), shevelev@bionet.nsc.ru (O.B.S.), zavjalov@bionet.nsc.ru (E.Z.)

* Correspondence: t_godovikova@mail.ru; Tel.: 8-383-3635183

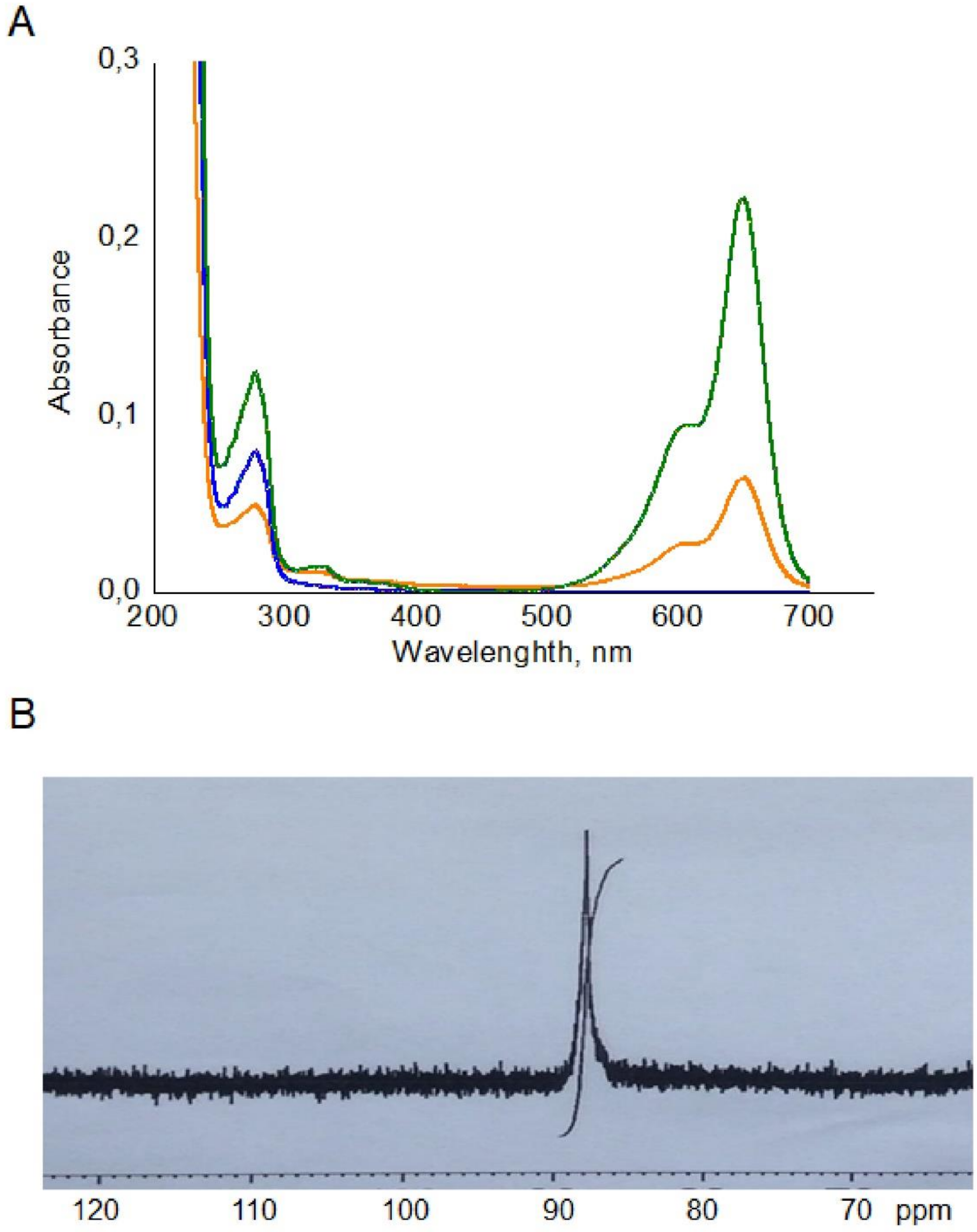

Figure. S1. Characterization of multifunctional human serum albumin-therapeutic conjugate HSA-Cy5-HcyTFAc-$B_{12}H_{12}$. A: UV-vis spectra of HSA homocystamides in PBS buffer, pH 7.4. HSA – blue; HSA-Cy5-HcyTFAc – green, HSA-Cy5-HcyTFAc-$B_{12}H_{12}$ – orange. B: ^{19}F NMR spectrum (at 282.4 MHz) of HSA-Cy5-HcyTFAc-$B_{12}H_{12}$ conjugate (0.6 mM) in PBS buffer (pH 7.4, to provide deuterium lock, D_2O was added to 20% of the total volume) at 37°C. The chemical shifts are referred to the resonance of C_6F_6 at 0.0 ppm.

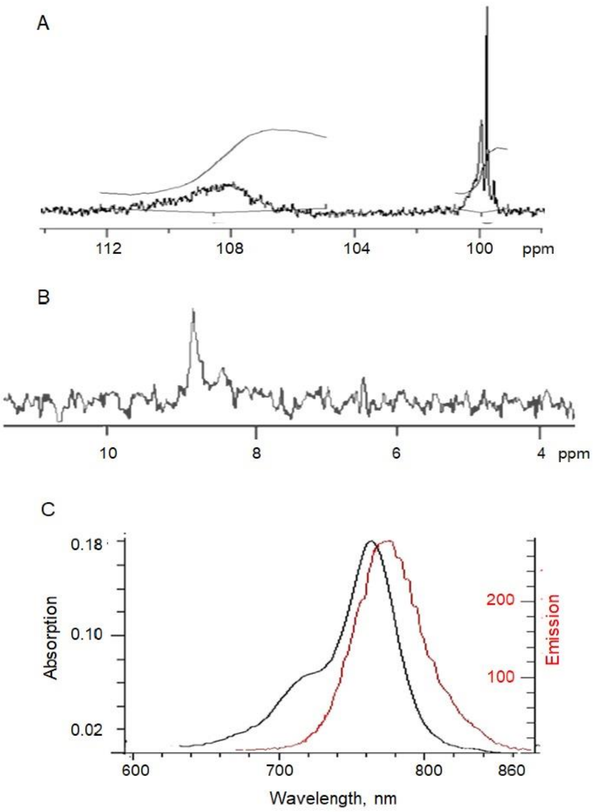

Figure S2. Characterization of multifunctional human serum albumin-therapeutic conjugate PFT-Hcy-HSA-Cy7-pTFT. A: ^{19}F NMR spectrum (at 282.4 MHz) of PFT-Hcy-HSA-Cy7-pTFT conjugate (0.6 mM) in PBS buffer (pH 7.4, to provide deuterium lock, D_2O was added to 20% of the total volume) at 37°C. The chemical shifts are referred to the resonance of C_6F_6 at 0.0 ppm. B: ^{31}P NMR spectrum (at 121 MHz) of PFT-Hcy-HSA-Cy7-pTFT conjugate (0.6 mM) in D_2O at 37 °C. C: Absorption (black) and Emission (red) spectra of PFT-Hcy-HSA-Cy7-pTFT (10 µM) recorded in PBS buffer (pH 7.4) at 37 °C.
